# Supplementary material for: Protocol for VIVALDI social care: Pilot study to reduce infections, outbreaks and antimicrobial resistance in care homes for older adults
Source: NIHR Open Res. 2024 Feb 15;4:4. [Version 1] doi: 10.3310/nihropenres.13530.1 (PMC11319897; doi:10.3310/nihropenres.13530.1)
Supplement: Peer review history

# Reviewer report: Reviewer response for version 1

**DOI:** 10.3310/nihropenres.14685.r31208

**Published:** 2024-05-24

**Authors:** Krystal Warmoth

**Affiliations:** 1 University of Hertfordshire, Hatfield, England, UK

This article presents the protocol for the VIVALDI social care pilot study which aimed to determine the feasibility of establishing a database of care homes to measure the frequency of infections. This will be undertaken by collecting data from residents and linking it to existing healthcare datasets. An anonymous database will be created for future use by researchers.  Overall, the article is well-written and clear. The methods seem appropriate. The work is strengthened by its involvement and engagement of key stakeholders as well as building on the learnings from the COVID-19 pandemic.

 Will the care homes that take part be given any incentives, rewards, or payment for taking part in the study or database? Please clarify and expand if so or if not.

 It would be helpful to know more about Engagement & Communications workstreams, especially as they will be used for dissemination of the findings and outputs and how they differ from the other involvement activities.

 More details about how the study outcomes will be shared with participants at the end of the study. Will this be done by communications and outputs shared with the participating care homes or only accessible via the website? Please clarify.

Is the study design appropriate for the research question?

Yes

Is the rationale for, and objectives of, the study clearly described?

Yes

Are sufficient details of the methods provided to allow replication by others?

Yes

Are the datasets clearly presented in a useable and accessible format?

Not applicable

Reviewer Expertise:

care home research, frailty, multimorbidity

I confirm that I have read this submission and believe that I have an appropriate level of expertise to confirm that it is of an acceptable scientific standard.

# Reviewer report: Reviewer response for version 1

**DOI:** 10.3310/nihropenres.14685.r31512

**Published:** 2024-05-21

**Authors:** Kin On Kwok

**Affiliations:** 1 The Chinese University of Hong Kong, Hong Kong, Hong Kong

The VIVALDI Social Care Project aims to establish a research database to support large-scale studies on infections and antimicrobial resistance (AMR) in older adults in English care homes. The project involves extensive stakeholder engagement, including residents, relatives, care home staff, and providers, achieved through various media and events. Two working groups have been formed to address engagement, communication, data sharing, and governance. The study will focus on care homes using digital social care records (DSCR) and has partnered with researchers and organizations to develop engagement materials and activities. 

 The study will recruit residents from participating care homes who have not opted out of data collection, aiming for inclusivity and generalizability. Due to cognitive impairments among many residents and staffing challenges, the study seeks Section 251 support to collect data without individual consent, with opt-out options available. Recruitment and engagement efforts have already garnered interest from over 800 care providers. The study will begin in January 2024, with data collection continuing for up to a year, and aims to create a robust database for future research on infection control and AMR in care homes.

 The objective and rationale of this study are clearly outlined. The study will be completed within 15 months from January and data analysis will be undertaken during that period.

 There are a couple of concerns.

 First, given the short timescales, while I understand this is a pilot study, I wonder if 15 months is sufficient to complete the entire process of testing the feasibility of establishing sentinel surveillance and creating a database with a targeted sample size. although the study provides an opt-out mechanism, ensuring that all residents are adequately informed and able to opt out if they choose could be challenging, particularly for the majority of those with cognitive impairments. I am not sure the process is sensitive to the number of subjects opting out of the study. 

 Second, collecting data without individual consent, even with Section 251 support, may raise privacy concerns among residents, relatives, and care home staff. This could affect trust and willingness to participate.

 Third, the materials will be co-developed with the study's engagement working group, which includes relatives, care home staff, and providers. I am concerned about varying levels of dedication to the program and the diverse backgrounds of the participating individuals affecting the context and consistency of the development process. 

 Finally, there's a concern that rushing the finalization of the dataset used to analyze the incidence of antimicrobial resistance (AMR) and its correlation with antimicrobial prescribing practices in care home residents might compromise its accuracy and completeness. It might be more effective to allocate a non-overlapping period (say additional 1 to 2 months on the existing schedule) with data collection specifically dedicated to wrapping up the findings and facilitating dissemination.

 The study design is appropriate. Strengths include extensive stakeholder engagement, clear inclusion and exclusion criteria, and a focus on digital social care record (DSCR) users. The partnership with organizations and researchers enhances credibility, and the opt-out mechanism aims for inclusivity. Excluding care homes with paper-based systems may limit generalizability of the current study although the electronic system will become available shortly, and sustaining engagement with a large number of stakeholders may be challenging.

 If the format of the sample final dataset can be provided, it will be easier for other populations to replicate or tailor-made the same system.

Is the study design appropriate for the research question?

Yes

Is the rationale for, and objectives of, the study clearly described?

Yes

Are sufficient details of the methods provided to allow replication by others?

Yes

Are the datasets clearly presented in a useable and accessible format?

Not applicable

Reviewer Expertise:

I am devoted to better infection control in vulnerable healthcare settings, such as residential care homes for the elderly, and to providing timely public responses to emerging and reemerging infectious diseases. My research interests are antimicrobial resistance (AMR), SARS-CoV-2, influenza, and Monkeypox.

I confirm that I have read this submission and believe that I have an appropriate level of expertise to confirm that it is of an acceptable scientific standard.

# Reviewer report: Reviewer response for version 1

**DOI:** 10.3310/nihropenres.14685.r31505

**Published:** 2024-05-02

**Authors:** Max Geraedts

**Affiliations:** 1 Philipps University Marburg, Marburg, Germany

The data to be supplied daily by the care homes to a secure environment within NHSE includes data on hospital admissions and emergency department visits, time and cause of death, biological and microbiological laboratory data and antibiotic prescriptions for each resident.

 The authors conclusively justify the need to establish such a database based on the experience gained during the COVID-19 pandemic in the UK. The study protocol clearly describes the careful involvement of patients, their relatives and the public in the development of the study design. The study setting, the inclusion and exclusion criteria, the timeline and the study objectives are explained very well. The data collection itself as well as the data management and governance of the study are also explained in sufficient detail. The study, in particular the waiver of consent for data collection for each individual subject, has already been approved by ethics committees. Finally, the plan for disseminating possible results of the study and the conclusions that may be drawn from the study are also explained.

 Overall, the authors present a very comprehensible study protocol, which in my opinion could only be improved in a few places. Firstly, it could be explained in more detail how the bias in study participation is dealt with, which is associated with the decision to include only those facilities that have electronic documentation (DSCR). Secondly, it should be made clearer what information about the care homes themselves should be included in the study. If, as explained in the study objectives, the aim is to develop options for preventing or reducing infections on the basis of the study, it should also be possible to analyze the relationship between the structural characteristics of the care homes (e.g. staffing and staff qualifications) and these outcomes. Thirdly, there is no explanation of how the findings of this pilot study will be incorporated into a definitive study. So what findings would lead to the data collection not being continued? Or how should it be ensured that a larger number of care homes, which are then also as representative as possible for the UK, participate in the data collection in future?

Is the study design appropriate for the research question?

Yes

Is the rationale for, and objectives of, the study clearly described?

Yes

Are sufficient details of the methods provided to allow replication by others?

Yes

Are the datasets clearly presented in a useable and accessible format?

Yes

Reviewer Expertise:

health services research

I confirm that I have read this submission and believe that I have an appropriate level of expertise to confirm that it is of an acceptable scientific standard.
